# Human Genome Variation and the Concept of Genotype Networks

**DOI:** 10.1371/journal.pone.0099424

**Published:** 2014-06-09

**Authors:** Giovanni Marco Dall'Olio, Jaume Bertranpetit, Andreas Wagner, Hafid Laayouni

**Affiliations:** 1 Institut de Biologia Evolutiva, CSIC-Universitat Pompeu Fabra, Barcelona, Catalonia, Spain; 2 Institute of Evolutionary Biology and Environmental Studies, University of Zurich, Zürich, Switzerland; 3 The Swiss Institute of Bioinformatics, Lausanne, Switzerland; 4 The Santa Fe Institute, Santa Fe, New Mexico, United States of America; 5 Universitat Autonòma de Barcelona, Barcelona, Spain; University of Florence, Italy

## Abstract

Genotype networks are a concept used in systems biology to study sets of genotypes having the same phenotype, and the ability of these to bring forth novel phenotypes. In the past they have been applied to determine the genetic heterogeneity, and stability to mutations, of systems such as metabolic networks and RNA folds. Recently, they have been the base for reconciling the neutralist and selectionist views on evolution. Here, we adapted this concept to the study of population genetics data. Specifically, we applied genotype networks to the human 1000 genomes dataset, and analyzed networks composed of short haplotypes of Single Nucleotide Variants (SNV). The result is a scan of how properties related to genetic heterogeneity and stability to mutations are distributed along the human genome. We found that genes involved in acquired immunity, such as some HLA and MHC genes, tend to have the most heterogeneous and connected networks, and that coding regions tend to be more heterogeneous and stable to mutations than non-coding regions. We also found, using coalescent simulations, that regions under selection have more extended and connected networks. The application of the concept of genotype networks can provide a new opportunity to understand the evolutionary processes that shaped our genome. Learning how the genotype space of each region of our genome has been explored during the evolutionary history of the human species can lead to a better understanding on how selective pressures and neutral factors have shaped genetic diversity within populations and among individuals. Combined with the availability of larger datasets of sequencing data, genotype networks represent a new approach to the study of human genetic diversity that looks to the whole genome, and goes beyond the classical division between selection and neutrality methods.

## Introduction

Genotype networks are a concept used in the field of systems biology to study the “evolvability” or “innovability” of a set of genotypes having the same, broadly defined, phenotype, such as viability, and to determine whether a given phenotype is robust to mutations [1], [2]. They have been used to study the evolvability of metabolic networks in simple organisms, by identifying how much a metabolic network can be altered without losing the ability of surviving using a given carbon source [3]–[7]. Similarly, they have been used to study the ability of a metabolic network to “evolve” a new phenotype, such as the ability of surviving on a new carbon source [8]. Genotype networks have also been used to study the robustness of RNA folds and protein structures, evaluating how many mutations can be accumulated in a sequence without losing the secondary structure [9]–[11].

The concept of genotype network is derived from the metaphor of protein space proposed by Wright [12], and adapted by Maynard Smith [13], a representation of all possible protein sequences as a framework to describe how evolutionary processes take place. This sequence space is explored by evolving populations, which, mutation after mutation, and through generations of individuals carrying similar sequences, reach proteins of maximal adaptive value. Although genotype networks have also been referred to as neutral networks [14], [15], we here prefer to use the term genotype networks, because we do not have any information on the phenotype of the sequences we study (the individuals of the 1000 Genomes dataset are anonymous), and we do not know whether all the nodes in a network are effectively neutral with respect to fitness.

Genotype networks are also at the base of a model proposed to reconcile the two neutralist and selectionist schools of thoughts in evolutionary biology [16]. According to this model, evolution is characterized by cycles of “neutral” evolution, in which populations accumulate neutral or even slightly deleterious mutations, followed by beneficial mutations, which can sweep through a population and thus allow a new repertoire of genotypes to accumulate in the population. The set of genotypes accumulating in a population during a cycle of neutral evolution lie on the same genotype network, and beneficial mutations are events that allow a population to switch from one genotype network to another. Under this model, even negative or neutral mutations can have a beneficial effect in the long run, as they allow a population to explore genotype space and increase the chances of finding a beneficial mutation [16].

In general, genotype networks are defined in relation to a given phenotype. For example, they can be used to compare the genetic variability of individuals with a genetic disease against a control dataset, or to study the genetic variation behind phenotypic traits like lactose tolerance or eye color. However, in the current work we present only a coarse-grained genome-wide analysis of how the properties of a population sample of a genotype network are distributed along the genome, defining the phenotype as viability, i.e., the mere “presence” of a genotype in any of the individuals of the 1000 Genomes dataset. We performed coalescent simulations to predict whether the sample size of the 1000 Genomes is representative of variation in real populations, and to verify how many samples are needed to represent networks of a given size. The genome-wide scan presented here can form a basis for future applications of genotype networks, and will permit better use of genotype networks to understand genome variation.

### Description of the genotype networks method

A genotype network is a graph whose nodes are genotypes such as DNA sequences (or, in our case, short haplotypes of Single Nucleotide Variants), and where two sequences are connected by an edge if they differ in a single nucleotide, corresponding to a single mutational step [1].

**Figure 1:**
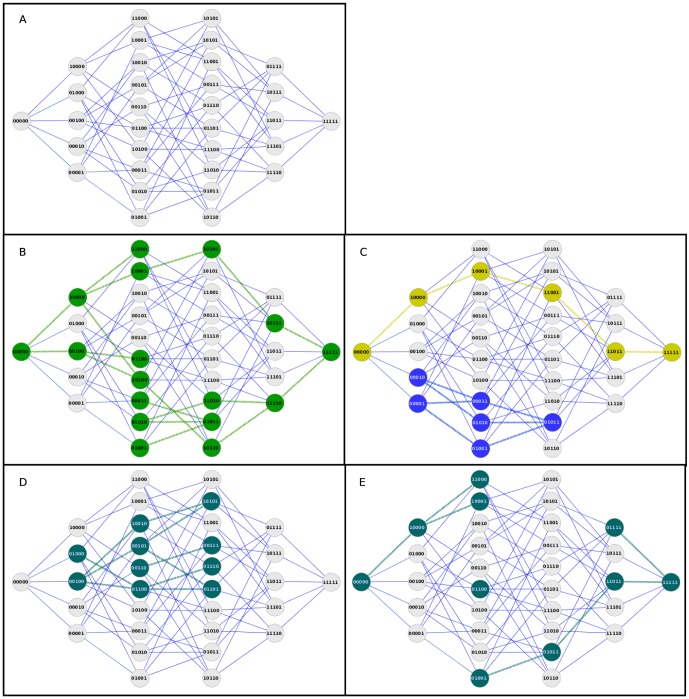
Examples of genotype networks and their properties. **A.** Representation of the Genotype Space for a region including 5 loci or Single Nucleotide Variants (SNVs). The space of all possible genotypes is represented as a Hamming graph (whole network). Each node represents one possible genotype, and each edge represents that the two nodes connected have only one difference. **B.** Example of genotype network. On top of the Genotype Space, we mark the genotypes observed in a population, and define it as the genotype network of that population (green nodes). **C.** Genotype networks of two populations (yellow and blue). The green population has a large average path length and diameter, while the blue population has a short average path length and diameter. **D.** Genotype network of a population having a high average degree and only one single component. **E.** Genotype network of a population having low average degree and many fragmented components.

To better understand the concept of genotype network, it is useful to introduce the notion of a genotype space, defined as the set of all possible genotypes in a region of the genome. For example, Figure 1A shows the genotype space of a region of five contiguous Single Nucleotide Variants (SNVs). Each node in Figure 1A represents one possible genotype as a string of “0”s and “1”s, where the “0”s represent the reference allele, and the “1”s represent the alternative allele. In this space, two genotypes are directly connected if they differ only in a single nucleotide, e.g. the nodes “00000” and “00001” are connected.

In empirical data, populations usually occupy only a small portion of an entire genotype space. As an example, in Figure 1B, we marked in green all the genotypes that are observed at least once in a hypothetical population. We define the genotype network of this population as the portion of genotype space occupied by the population. The method we propose is based on comparing the properties of a genotype network to the whole genotype space, and asking question about its extension in this space, and how tightly connected its nodes are.

In particular, we focus on two classes of attributes of genotype networks. The first class is suitable to understand how a network is distributed throughout this space. The second class of attributes relate to the connectivity of the network, and has implications on the network's robustness when a point mutation appears.

#### Extension and heterogeneity of the genotype network

The first two attributes of interest are the number of vertices and the average path length of a genotype network. These attributes allow one to study how far a network extends through genotype space, and how heterogeneous or genetically distant the genotypes in a population are.

As an example of how two populations might differ in these properties, Figure 1C shows the genotype networks of two hypothetical populations, one having high average path length (yellow nodes), and the other low average path length (blue nodes). Even though the two populations contain the same number of distinct genotypes (vertices), the yellow population is genetically more heterogeneous, and its individuals are genetically more diverse, than the individuals of the blue population. Specifically, the yellow population contains individuals that have genetically very distant genotypes, such as “00000” (all loci having the reference allele) and “11111” (all loci having the alternative allele), while in the blue populations, the genetically most distant individuals have at most two allelic differences (e.g. “00001” and “01001”). Thus, the distance of the vertices and the average path length of the genotype networks of these two populations indicate that they adiffer in how far they extend through the space.

#### Robustness and stability of the genotype network

Two other attributes of interest are the number of components and the average degree of genotypes in a genotype network. In other literature on genotype networks, these attributes have been used as measures of robustness to mutations [5], [17]. In this work, since we do not have detailed phenotypic information, we prefer to speak about the stability of a genotype network in a genomic region to mutations.

Figures 1D and 1E show two hypothetical genotype networks that differ in the number of components and their average degree. Both networks occupy the same number of nodes, but on average the nodes of the genotype network of Figure 1D (only the blue nodes) have more connections than the nodes in the network of Figure 1E. Specifically, in the network of Figure 1D, most nodes are connected to at least three other nodes, whereas the network in Figure 1E is much more fragmented, as most nodes have only one or two connections, and some groups of nodes are not even connected.

The biological significance of these attributes can be seen by considering the effect of a random point mutation on a network node (genotype). If we randomly select a genotype and mutate one of its nucleotides, the resulting genotype will be one of its neighbors in the network, because, by definition, nodes in a genotype network are neighbors if they differ in a single nucleotide. As the connectivity of a genotype network increases, the chances increase that a mutant genotype will be a genotype that is already part of the network. For example, if we take the node ‘01100’ in Figure 1D or 1E, and simulate a random point mutation, the result will be one of the five genotypes ‘01000’, ‘00100’, ‘11100’, ‘01110’, or ‘01101’. In Figure 1D, four out of these five genotypes already belong to the genotype network, so a point mutation is not likely to create genotypes outside the genotype network. In Figure 1E, however, all nodes connected to the original genotype do not belong to the genotype network, so any point mutation must create a genotype outside the network. Thus, we can interpret genotype networks with few connected components and with high average degree as more stable to mutations than other types of genotype networks.

## Results

### Genome-wide distributions

We executed a genome-wide scan of genotype networks for the 1000 Genomes dataset, producing an overview of how the number of vertices, the average path length, the number of components, and the average degree of the genotype networks are distributed on the human genome. The scan is implemented as a series of sliding windows that subdivide the genome into overlapping regions of 11 SNVs (see Methods). The results are available as a UCSC browser custom track hub, accessible at http://genome.ucsc.edu/cgi-bin/hgTracks?db=hg19&hubUrl=http://bioevo.upf.edu/~gdallolio/genotype_space/hub.txt. Raw data can be downloaded using the UCSC Tables function or forwarded by request.

Table 1 presents an overview of the genomic regions having the highest values of each of the network properties we calculated. Interestingly, most of these top regions are associated with genes involved in acquired immunity, such as HLA and MHC genes. In particular, the three regions with the highest average path length belong to HLA genes (HLA-DRB1, HLA-DRB5 and HLA-DQA1), while a region in HLA-DPA1 shows an exceptionally number of connected components. Moreover, if we divide the number of vertices by the number of components, we find that two regions related to the MHC I complex, MICA and MICB, have especially large components.

**Table 1:** Regions showing top scores in the genome.

region	criteria	Closest	Distance to	Description of	2nd closest	Description of 2nd closest
		gene	closest gene	closest gene	gene	gene
chr2:91959344-91968231	high number of components	GGT8P	inside gene	pseudogene		
chr6:33037767-33038449	high number of components	HLA-DPA1/HLA-DPB1	inside gene	Homo sapiens major histocompatibility complex, class II		
chr:7203189-7420319641	high number of components	ITGB8	50,684 bp	integrin	HLA-DPA1/	major histocompatibility
					HLA-DPB1	complex, class II
				praja ring finger 2,		
chr5:108634323-108635534	high average degree	PJA2	34,876 bp	E3 ubiquitin protein ligase	AK021888	unknown function
chr8:25935936-25937929	high average degree	EBF2	inside gene	early B-cell factor 2		
				Homo sapiens		
chr6:32507854-32508257	high average path length	HLA-DRB1	inside gene	major histocompatibility complex, class II		
				Homo sapiens		major
chr6:32568909-32569343	high average path length	HLA-DRB5	11,297 bp	major histocompatibility complex, class II	HLA-DQA1	histocompatibility complex, class II
				Homo sapiens		
chr6:32611264-32611586	high average path length	HLA-DQA1	inside gene	major histocompatibility complex, class II		
chr3:36921415-36921688	high number of vertices	TRANK1	inside gene	tetratricopeptide repeat and ankyrin repeat Containing 1		
chr4:9176678-9178624	high number of vertices	C9JJH3	33,759 bp	Deubiquitinating enzyme	LOC650293	transmembrane helix receptor
chr8:35105546-35106981	high number of vertices	UNC5D	inside gene	receptor of netrin involved in nervous system		
chr4:9200148-9202368	few components, but large number of vertices	USP17L10	10,015 bp	Deubiquitinating enzyme		
chr6:31357915-31358747	few components, but large number of vertices	MICA	8,814 bp	MHC class I polypeptide-related sequence A	HLA-B	major histocompatibility complex, class I
chr6:31455010-31456012	few components, but large number of vertices	MICB	6,646 bp	MHC class I polypeptide-related Sequence B	uc003ntm.3	HLA complex Group 26 (non-protein coding)

The higher genetic heterogeneity (in terms of the average path length and larger component size) of these regions involved in acquired immunity can be explained by their role in interacting with disease agents. The HLA and MHC regions are known for being among the most variable regions within human populations, and their function in interacting with pathogens greatly increases the genetic variability between individuals [18], [19]. The higher genetic heterogeneity of many regions involved in acquired immunity can be interpreted as a high capacity for finding novel responses to different classes of pathogens. In this sense, the exceptionally high number of components in the HLA-DPA1 region is a contrasting observation, as so many components indicate a very fragmented network. Maybe the diversity of this region is so high that our sample size is not able to capture it, thus identifying a fragmented network instead of a large connected component.

### Evaluating the effect of missing samples

A difficulty in applying genotype networks to SNV data is that one needs many individuals to correctly represent the genetic variation in real populations. If the number of individuals in a dataset is not enough, some genotypes or haplotypes may not be represented in the network, not because they are not present in the real population, but just because they are missing from the population sample. In particular, some properties such as average path length and average degree cannot be calculated properly (in mathematical terms) when there are too many missing nodes in a network, so it is important to understand the effect of sample size on our ability to infer genotype network properties.

**Figure 2:**
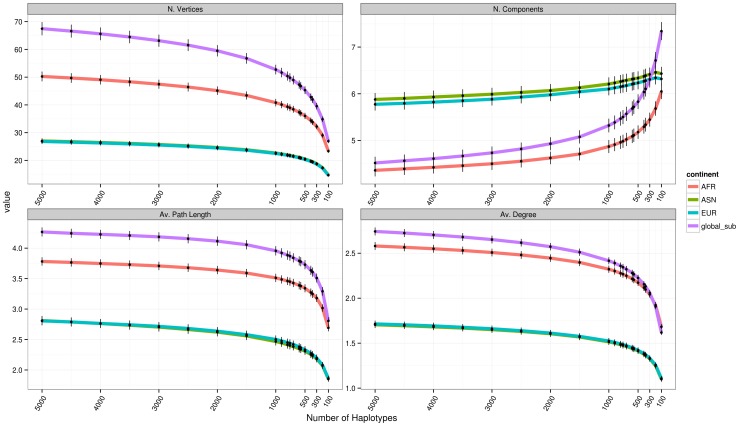
Distribution of Genotype network properties in a set of coalescent simulations, varying the number of samples used to construct the networks. We first simulated 15,000 haploid sequences (5,000 for each of an African, Asian and European population). Then, we randomly sampled a given number of haplotypes, from 5,000 to 100, and calculated the distribution of properties of genotype networks built using only the randomly sampled individuals. Networks are based on 11 SNVs, and each data point represents the average of five random subsamplings of the same size. Bars are too short to be visible for most data points, and show 6 times the standard deviation of the mean.

To evaluate the effect of missing haplotypes, we performed coalescent simulations including 5,000 haplotypes (2,500 diploid individuals) for each of the African, Asian and European populations, for a total of 15,000 haplotypes. From this simulated dataset, we successively sampled a number of randomly chosen haplotypes, with as few as 100 haplotypes (50 individuals) per population, and we observed how properties of genotype networks varied as we reduced this sample size. The results are shown in Figure 2. Each data point in the figure represent the average of 5 independent re-samplings of the same number of individuals, using networks of 11 SNVs.

Figure 2 shows that properties of genotype networks change nearly linearly for a sample size of 5,000 to 1,000 haplotypes, suggesting that for this range of sample sizes the effect of missing haplotypes is not very strong. Interestingly, the relative differences between populations remain the same, independently of sample size, e.g., European and Asian populations have similar values at different sample sizes, and the global population always has a higher number of vertices, greater average path length, and greater average degree than the three sub-populations. For lower sample sizes, from 1000 to 100 haplotypes (corresponding to 500 to 50 diploid individuals), the quantities we computed change more sharply, suggesting that the effect of missing samples is substantial and may cause wrong interpretation of the results. For example, for a sample of 300 haplotypes, the global population has more components than the African population, reversing the result observed for higher sample sizes. Overall, this analysis suggests that for networks of 11 SNVs, only observations based on more than 1,000 samples should be trusted.

### Correlation between Network Properties

**Figure 3:**
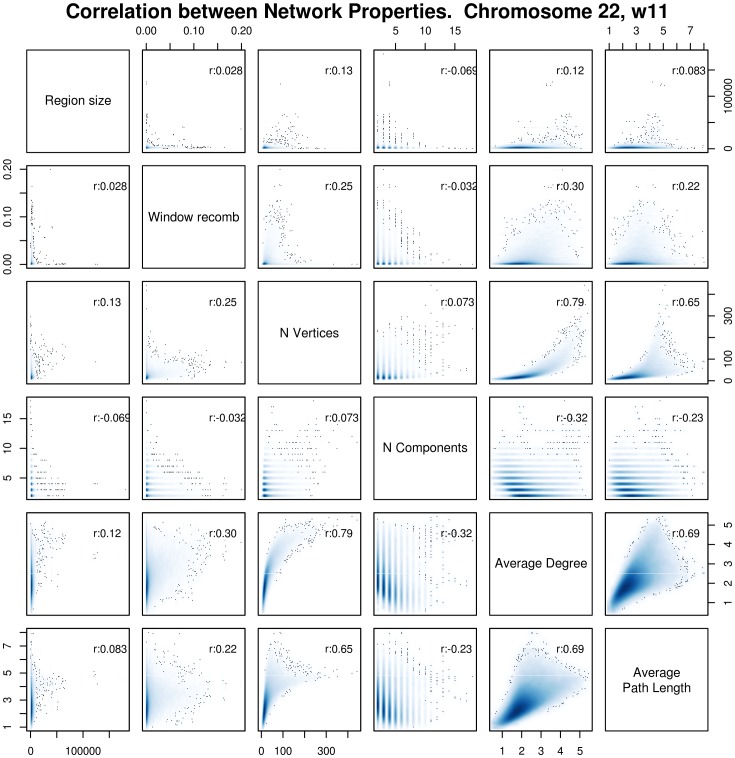
Correlation between Genotype network properties on chromosome 22. Each panel shows the pairwise distributions of two properties, one of each axis. For example, the two squares at the intersection of “Average Degree” and “Average Path Length” show the pairwise distribution of scores for these properties. The intensity of the blue shape is proportional to the density of points, while black dots represent outliers of the distribution, and facilitate the visualization of the limits of each distribution. Ideally, if there is a linear correlation between two properties, a linear plot should appear. The pairwise correlation coefficient is shown in all panels, in the right bottom corner. All correlations are significant (p value <0.05).

Figure 3 shows pairwise correlations between genotype network properties for data based on chromosome 22, also comparing them with region size and recombination rate. Each panel in the figure shows two properties, one on the X axis, and the other on the Y axis, as defined in the diagonal panels. For example, the bottom-left panel shows a pairwise comparison between region size (on the X axis) and average path length (on the Y axis), and also indicates that these properties are correlated with a Pearson coefficient of r = 0.089.

The first two rows and columns show the effect of region (window) size and of recombination rate on genotype network properties. The region size refers to the length, in base pairs, of the region occupied by a network spanning 11 SNVs (2–3 kb on average). The recombination rate is a measure of the average amount of recombination observed from the first to the last SNV in an 11 SNV window, and was obtained from the 1000 Genomes website. These two properties allow us to determine if we have to correct for these factors when comparing genotype network properties among different regions. Notably, there is a small but significant effect of recombination on the average degree (r = 0.34; P<1×10^−30^), on the number of vertices (r = −0.29; P<1×10^−30^), and on the average path length (r = 0.23; P<1×10^−30^). Given this relationship, we took into account recombination when comparing network properties across regions in some analyses (e.g. see “Genotype Networks of Coding and Non-Coding regions”).

The remaining panels in Figure 3 show the pairwise correlation between all other network properties. Some properties are clearly correlated. For example, the average degree and the number of vertices have a correlation coefficient of 0.80 (P<1×10^−30^), meaning that large networks also tend to have larger average degree. Notably, the correlation between these two properties increases if we use a logarithmic scale for the number of vertices (r = 0.89, p-value<1×10^−30^). Together with recent results that have demonstrated a similar correlation both in RNA models [20], and in models of protein complexes [21], this suggests that a logarithmic correlation between network size and degree may be a universal feature of genotype networks.

Figure 3 also shows that the average degree and the average path length have a correlation coefficient of 0.70 (P<1×10^−30^). This correlation may be caused by the fact that adding one node to a small network increases both the average path length and the average degree. Consistent with this notion, this correlation becomes weaker for larger networks (Figure 3).

### Genotype Networks of Coding and Non-Coding regions

We used the functional annotations from the 1000 Genomes website to determine whether the presence of a coding or of a non-coding SNV affects the properties of a genotype network. In particular, we restricted our analysis to all the SNVs having a functional effect (according to the ENCODE annotations [22]), and classified all networks into four categories, according to the effects of the SNVs included. The classes are:

- networks containing only coding SNVs;
- networks containing both coding and non-coding SNVs;
- networks containing only non-coding SNVs;
- networks containing only SNVs in intergenic regions, or for which no annotation is available, and which have no known functional effect (excluded from further analysis).

These annotations are obtained from the 1000 Genomes website, and are calculated using the Variant Annotation Tool [23], [24]. For simplicity, in the rest of the paper, we refer to these classes as “coding”, “both”, “noncoding”, and “no annotations”.

Since we had previously shown that the recombination rate is correlated with some network properties (see the section “Correlation between Network Properties”), we removed the networks with the highest recombination rate (more than 1 cM between the first and the last SNV of the window). Moreover, to compare networks based on windows belonging to different annotation classes, we applied an analysis of covariance, using the annotation category as a grouping variable, and the recombination rate as a covariable. This analysis aims at comparing the functional categories for genotype network properties taking into account the effect of recombination rate, and we performed it separately for each of our three subpopulations. Networks of the class “no annotations” (containing only SNVs for which no annotation is available) are excluded from this analysis, as a clear interpretation for this category cannot be provided.

Overall, the three classes of networks from our different subpopulations have different numbers of connected components, a difference that is significant between all three populations. Specifically, networks containing only coding SNVs have fewer connected components than networks comprised of non-coding SNVs (see Table S1). Moreover, networks containing both coding and non-coding SNVs have intermediate numbers of connected components. Thus, networks containing coding SNVs are more connected, while non-coding networks tend to be more fragmented. These differences hold in all pairwise comparisons between the coding and noncoding classes, even when a Bonferroni multiple testing correction is used (p<0.009 in all comparisons).

**Figure 4:**
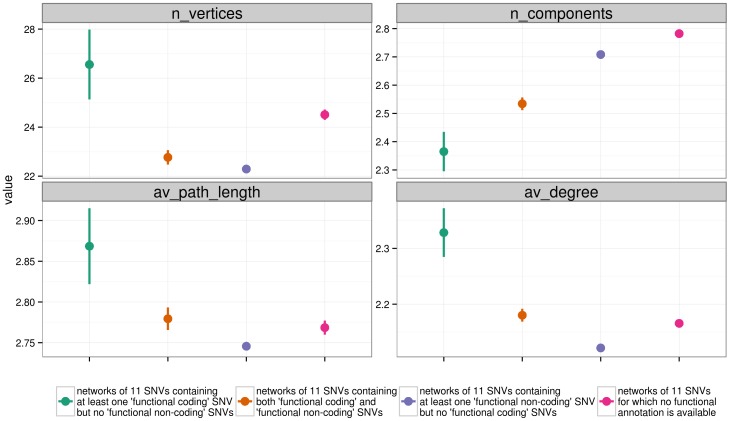
Distribution of network properties, comparing SNV functional annotations. Each point shows the mean +/−2 standard deviations of the mean for a class of networks.

Figure 4 shows the means of these and other network properties for chromosome 22 in the global population. Coding networks tend to have more vertices, greater average path length, and greater average degree than non-coding networks. Overall, these results show that coding regions are less fragmented (they have fewer components) than non-coding networks, but at the same time, they are more extended in genotype space (higher path length), and are more connected (higher average degree). It should be noted that even though the differences between annotation categories reach statistical significance in almost all comparisons and for almost all properties, the magnitude of the observed differences is small in general.

### Effects of a simulated selective sweep on genotype networks

**Figure 5:**
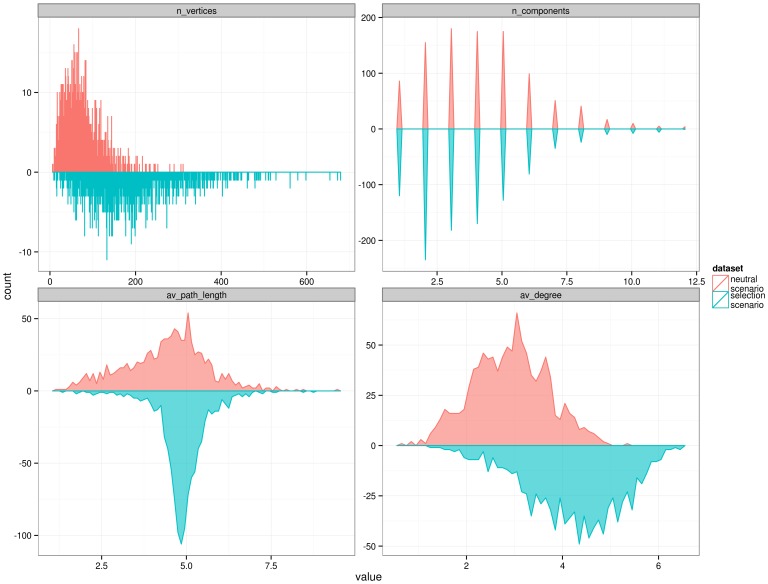
Distribution of network properties, comparing a dataset of neutral demography simulations against a scenario of selective sweep. Selection scenario simulates a recent selective sweep with a selection coefficient of 0.015, and a final frequency of 0.99. The networks included in this graph are calculated by merging the 5,000 haplotypes of the three populations simulated (African + European + Asians) into a global population, and calculating the genotype networks on all the 15,000 haplotypes together.

Figure 5 compares the distribution of Network Properties between the data from chromosome 22 and two simulated datasets, representing a neutral and a selection scenario. The neutral scenario is based on the known demography for the European, Asian and African populations [25]; The selection scenario is based on the same parameters as the neutral scenario, but adds a simulated selective sweep with a small selection coefficient (0.015), in which the selected allele reaches a final frequency of 0.99. We chose this scenario of weak selection, because it has recently been proposed that strong selection events were rare in our evolutionary history [26], [27]. This analysis shows that all four genotype network properties we consider differ between the neutral and selection scenarios. The least marked difference occurs in the number of components, where the selection simulations show a slightly lower number of components than the neutral scenario (Wilcoxon test: W = 575321.5, p-value  = 2×10^−9^). In contrast, the selection scenario leads to a higher number of vertices, average path length, and degree than the neutral scenario (p<10×10^−15^ for Wilcoxon test, for all the properties). In particular, the quantile-quantile plots (qqplots) shown in Figure S3 show that in the selection scenario the proportion of average path length values close to 4 is greater than in the neutral scenario. Together, these results indicate that after a selective sweep genotype networks tend to be both more stable and connected (lower number of components and higher average degree) and at the same time more extended in genotype space (higher number of vertices and average path length).

## Discussion

### Strategies to apply genotype networks to next generation sequencing data

So far, genotype networks have not been applied extensively to population genetics data. The main reason is that doing so requires very large datasets, on the order of thousands of sequences or more. Even in the work presented here, we limited our analysis to regions spanning 11 SNVs, because according to coalescent simulations, the number of samples in the 1000 Genomes dataset is only large enough to reconstruct networks of this size with reasonable accuracy. In the future, larger datasets will make it possible to analyze larger regions, but for the moment, the limitation of small sample size can be overcome by constructing genotype networks through a sliding window approach, as presented in this paper. Thanks to this approach, it is possible to compare regions or genes of different size, by comparing the distributions of network properties among all windows.

Another difficulty in applying genotype networks to SNV data is that some network properties are associated with the recombination rate. In particular, network degree and number of vertices increase as the recombination rate in a region increases. Moreover, one would expect that a recombination event can fragment a genotype network, creating networks divided into multiple unconnected components. In our current analysis, we do not distinguish if the fragmentation of a network is caused by recombination, population demography, or other factors. This difficulty could be partially solved by removing the windows that show higher recombination rates (likely to contain recombination hotspots), and by applying multivariate analysis, using recombination as a covariable.

### Genotype Networks and Human Genome Variation

In this work we presented a genome-wide scan of how the properties of genotype networks are distributed in the human genome, using data from the 1000 Genomes project.

One observation is that there are small, but significant differences between networks of coding regions and those of non-coding SNVs. First of all, networks including coding SNVs tend to be less fragmented (lower number of components) than non coding networks. This result is perhaps intuitive, as we can expect that coding regions, being exposed to higher selective constraints than coding regions, would accumulate fewer mutations, and therefore have less fragmented networks. Moreover, we can expect that mutations in coding regions would accumulate at a slower pace and fall within few mutational steps from the genotypes of the previous generations, while mutations in non-coding regions would accumulate faster and fall more often within more than one mutational step from the previous genotypes. This would lead to an higher fragmentation of genotype networks in non-coding regions, especially in the present work, where networks are defined using a mutational distance of one.

Secondly, our results showed that coding networks are both richer in genotype diversity (higher average path length), and at the same time more stable to mutations (higher average degree). This result can be a consequence of their lower fragmentation. If a network is fragmented into multiple components, long paths of genotypes connected by one single mutational step each become less likely. Similarly, genotypes of more fragmented networks are less likely to be connected to other genotypes in the network, and this reduces the average degree of the network. It is also possible that the higher fragmentation observed is due to undersampling. More precisely, non-coding regions would require a higher number of samples to be fully reconstructed, compared to coding regions, such under-sampling could affect non-coding regions more than coding regions.

In addition, our analysis on simulated sequences showed that regions simulated under a selection scenario have also fewer components, but more vertices, greater average path length, and greater average degree, compared to regions exposed to neutral drift. This suggests that a selection event may have a direct impact on the structure of a genotype network. Genotype networks may help identify potential past selection events.

### Future Directions

As the cost of genome sequencing will decrease in the next years, and as larger datasets of sequences will become available, genotype networks may become useful tools to understand genome variation. One could employ them to analyse datasets of case and control individuals, and to better understand the genetic variation associated with a disease. In this case, genotype networks could be defined in reference to the presence or absence of a given disease, which is a more specific phenotype than the one used in this paper. Such an analysis may even allow us to learn how to identify genomic variants associated with potential diseases. However, doing so will require very large datasets of case/control individuals.

In the present work, we showed how genotype networks can be applied to study intra-specific variation, in particular in the human genome. We provided a few examples of how to use genotype networks to study this type of variation. We showed that it may be necessary to take into account the effect of recombination, and that some genotype network properties are associated with one another in empirical data. Moreover, we provided a description of the background distribution of these properties in the whole genome, and how they vary among coding and non-coding regions. The work presented here may constitute a starting point for applying genotype networks to study genome variation more extensively.

## Materials and Methods

### Genotype Datasets and Individuals

We downloaded Single Nucleotide Variant (SNV) genotype data from the Phase I release of the 1000 Genomes dataset (ftp://ftp.1000genomes.ebi.ac.uk/vol1/ftp/phase1/analysis_results/integrated_call_sets/) [28] on January 2013 (revision 2ff9d3af6cde in the repository, see “Reproducibility of the study”). Using the suite vcftools [29], we removed all the SNVs having a minor allele frequency in the global population lower than 0.01, and a coverage lower than 2-fold. We considered only phased SNVs, and did not analyze chromosomes X and Y. A total of 11,684,193 SNVs passed this filtering, with an average of one SNV every ∼250 bases.

From the 1000 Genomes dataset, we excluded all 242 American individuals (labels MXL, CLM, PUR, and ASW on the 1000 genomes website). One reason to exclude these populations is that it facilitates the comparison with the coalescent simulations, as no accurate demographic model for these populations is available [23]. A second reason is that, based on a principal component analysis (not shown), these individuals appeared to be genetically admixed with individuals from three other continents. The resulting dataset is composed of 850 individuals, or 1,700 haploid sets (chromosomes) grouped into individuals from three continents, African (AFR), Asian (ASN), and European (EUR). The African group includes 185 individuals (Yoruba from Nigeria and Luhyia from Kenya); the Asian group includes 286 individuals (Chinese from Beijing and South China, plus Japanese); the European group includes 379 individuals (Utah residents, Finland, Great Britain, Spain, Italy).

For the analysis of coding/non coding regions (see “Genotype Networks of Coding and Non-Coding regions) we used the functional annotations on SNVs from the 1000 Genomes ftp site (ftp://ftp.1000genomes.ebi.ac.uk/vol1/ftp/phase1/analysis_results/functional_annotation/annotated_vcfs). These annotations were generated by the 1000 genomes consortium, using the Variant Annotation Tool [23], [24]. The category of “functional coding” SNVs includes SNVs that are in a protein coding region, and that are transcribed and included in the mature transcript. The category of “functional non-coding” SNVs includes all the SNVs in non-coding regions that lie in transcription factor binding sites and in UTR regions, plus all the SNVs in regions that are transcribed but do not have any function, such as those in pseudogenes. All the other SNVs are included in a category called “no functional effect known”, which includes all the SNVs for which no annotation is available. Intronic SNVs are included in this latter set, if there is no evidence for any functional effect. This last category of “no functional effect known” has been excluded from later analysis, as no clear interpretation of this set was possible.

### Construction of Genotype Networks

Genotype Networks are computed using a customized version of Networks, a software produced by our group [29]. VCF2Networks allows to parse a Variant Call Format (vcf) file [30], generate a genotype network from it, and calculate network properties. The igraph library [31] and its python bindings are used to represent graphs and to calculate network properties.

Figure S1 schematically shows the protocol used to convert a vcf file to a genotype network. The first step is to apply the Minor Allele Frequency filter of 0.01 described above, and to remove all SNVs that have unphased data, or that are triallelic. Then, to generate the networks, we consider the two haplotypes of each individual as separate entities. Each genotype is encoded as a binary string, where “0” represents the reference allele, and “1” the alternative allele, using the annotations from the downloaded vcf files (triallelic loci are not included in the 1000 Genomes dataset). After encoding all the distinct genotypes observed in a population, we build a network in which each node represents one genotype, and an edge connects two nodes if they differ in a single allele between each other, i.e., if the Hamming distance between their binary string representations is equal to one.

### Description of Network Properties

Among the properties whose calculation is implemented in the tools VCF2Networks, we calculated the following for this contribution: the number of vertices, the average path length, the number of components, and the average degree. Here is a short description of how each of these properties is computed.

The number of vertices is equivalent to the number of distinct genotypes present in a population. Notably, due to the definition of a genotype network used here, the number of vertices is equivalent to the Dh statistics described by [32]. As an example, the network in Figure 1B has 17 vertices, while both networks in Figure 1C have exactly six vertices. The average path length is the average of all possible shortest paths between pairs of genotypes in a network, and it corresponds to the average number of single nucleotide changes that it takes to move from any node in the network to another. In the example of Figure 1C, the yellow network has an average path length of 2.33, and the blue network has an average path length of 1.67. Genotype networks of populations that have explored a greater portion of genotype space would have a higher number of vertices and a higher average path length.

A connected component of a graph is a subgraph in which each pair of nodes is connected through a continuous path of edges. The number of components of a network is the number of such subgraphs. For example, the network in Figure 1B contains a single component, while the network in Figure 1E contains three connected components, as there are three disconnected groups of nodes. The degree of a node is the number of its neighbors, i.e., nodes connected to it by a single edge. For example, in Figure 1D, the node “01000” has a degree of one, as it is connected to only one other node, while the node “01100” has a degree of four, as four edges emanate from it. The average degree of a network is the average of the degrees of all the nodes in the network: in Figure 1D, it is 2.20. Nodes without edges are called isolated and have degree zero. For networks with more than one component and some isolated nodes (e.g., Figure 1E), all components, including those comprising only a single, isolated node, are included in the calculation of the average degree. For example, the network in Figure 1E has an average degree of 1.54. As explained in the Introduction, we interpret the number of components and average degree as a measure of the stability of a genotype network to point mutations.

### Sliding windows approach

In order to compute genotype networks in a genome-wide scan, we divided the genome into contiguous and overlapping windows of 11 SNVs, building networks based on this fixed size. We chose a window size of 11 SNVs after having tested different window sizes on chromosome 22. More specifically, Figure S2 shows how the properties of the genotype networks of chromosome 22 vary with window size. In particular, for a window size of 11 SNVs, the networks of all the African, Asian and European populations have a similar number of components, while for larger sizes these three populations start to differ in this respect. Having similar component numbers for all populations is important because in mathematical terms, calculating properties such as the average degree of networks may lead to incomparable results when based on different numbers of components.

### Calculation of Genome Wide top scores and filters

To calculate which region showed the highest values for each network property in the whole genome, we first removed all networks for regions with low quality sequence or included alignment gaps. To do so, we filtered out all networks in which at least one SNV intersected one base with the “Gap” track in the UCSC Genome Browser (http://genome.ucsc.edu/cgi-bin/hgTrackUi?g=gap, last modification 2009-03-08). We also removed all regions corresponding to centromeres, to Giemsa band neighbors of the centromers, and to the first and last Giemsa bands of each chromosome, corresponding to telomeres. Then we applied a filter based on the quantile distribution of the remaining scores and on manual inspection (to remove possile artifacts) to identify only the top-scoring regions for each network property.

### Simulations

We implemented two sets of coalescent simulations, one based on the known demographic model (thus representing neutral evolution) and one simulating a selective sweep. We performed these simulations using the COSI software [25], version 1.2.1. Specifically, we simulated 3 populations (African, European, and Asian) of 5,000 individuals each, under the known demographic models for them [25]. The parameters used for the simulations represent an out-of-Africa migration event 3,500 generations ago, followed by a split between European and Asian populations 2,000 generations ago, and, in the case of simulations with selection, a selective sweep in which the selected variant has a selection coefficient of 0.0150 and a final frequency of the selected allele of 0.99. The exact parameters used for the simulations are available in the repository of this project (https://bitbucket.org/dalloliogm/genotype_space). After performing the simulations, we applied a filter of Minor Allele Frequency >0.01, removing all SNVs that had a low frequency in all three populations, i.e., we used the same criterion that we had used to filter the 1,000 Genomes data. These two simulated datasets allowed us to estimate the distribution of network properties under a well-defined demographic model, and to estimate the distribution of these properties for a larger sample size (5,000 chromosomes per population). Moreover, these simulations allowed us to evaluate the effects of a strong selective sweep on the properties of Genotype Networks.

### Reproducibility of the study, and other tools used

Following the best practices described in [33] the whole project presented in this manuscript, including the raw data, the scripts to produce plots and analysis, and a versioned log of all the commands used, are available at https://bitbucket.org/dalloliogm/genotype_space.

Figure 1 was generated using the Cytoscape software [34]. To manipulate genome-wide data, we used the bedops [35] and the bedtools [36] suites.

## Supporting Information

Figure S1 **Workflow used to calculate genotype network properties from a VCF file.**
(PNG)

Figure S2 **Distribution of genotype network properties in chromosome 22, changing the number of SNVs used to generate each network (window size), from 5 to 29 SNVs.** In order to have the same number of individuals in each population, each point is based on 5 samples of 370 haplotypes.
(PNG)

Figure S3 **Quantile-quantile plots of neutral vs selection simulations.** Only the networks of the global populations (African + European + Asians) have been included.
(PNG)

Table S1 **Wilcoxon test comparing coding and non coding networks.**
(DOC)

## References

[1] Wagner A (2011) The Origins of Evolutionary Innovations. Oxford University Press, USA.

[2] Manrubia SC, Cuesta JA (2010) Neutral networks of genotypes: Evolution behind the curtain. Arbor 186: 7 10.3989/arbor.2010.746n1253

[3] Wagner A (2009) Evolutionary constraints permeate large metabolic networks. BMC Evol Biol 9: 231 10.1186/1471-2148-9-231 19747381 PMC2753571

[4] Wagner A (2007) From bit to it: how a complex metabolic network transforms information into living matter. BMC Syst Biol 1: 33 10.1186/1752-0509-1-33 17663759 PMC1994685

[5] Matias Rodrigues JF, Wagner A (2009) Evolutionary plasticity and innovations in complex metabolic reaction networks. PLoS Comput Biol 5: e1000613 10.1371/journal.pcbi.1000613 20019795 PMC2785887

[6] Samal A, Matias Rodrigues JF, Jost J, Martin OC, Wagner A (2010) Genotype networks in metabolic reaction spaces. BMC Syst Biol 4: 30 10.1186/1752-0509-4-30 20302636 PMC2858107

[7] Dhar R, Sägesser R, Weikert C, Yuan J, Wagner A (2011) Adaptation of Saccharomyces cerevisiae to saline stress through laboratory evolution. J Evol Biol 24: 1135–1153 10.1111/j.1420-9101.2011.02249.x 21375649

[8] Barve A, Wagner A (2013) A latent capacity for evolutionary innovation through exaptation in metabolic systems. Nature 500: 203–206 10.1038/nature12301 23851393

[9] Ferrada E, Wagner A (2010) Evolutionary innovations and the organization of protein functions in genotype space. PLoS One 5: e14172 10.1371/journal.pone.0014172 21152394 PMC2994758

[10] Schultes EA, Bartel DP (2000) One sequence, two ribozymes: implications for the emergence of new ribozyme folds. Science 289: 448–452. 10903205 10.1126/science.289.5478.448

[11] Wagner A (2012) The role of robustness in phenotypic adaptation and innovation. Proc Biol Sci 279: 1249–1258 10.1098/rspb.2011.2293 22217723 PMC3282381

[12] Wright S (1932) The Roles of Mutation, Inbreeding, Crossbreeding and Selection in Evolution. Proc Sixth Int Congr Genet 01: 356–366.

[13] Maynard Smith J (1970) Natural Selection and the Concept of a Protein Space. Nature 225: 563–564 10.1038/225563a0 5411867

[14] Lipman DJ, Wilbur WJ (1991) Modelling neutral and selective evolution of protein folding. Proc Biol Sci 245: 7–11 10.1098/rspb.1991.0081 1682931

[15] Fontana W, Schuster P (1998) Shaping space: the possible and the attainable in RNA genotype-phenotype mapping. J Theor Biol 194: 491–515 10.1006/jtbi.1998.0771 9790826

[16] Wagner A (2008) Neutralism and selectionism: a network-based reconciliation. Nat Rev Genet 9: 965–974 10.1038/nrg2473 18957969

[17] Payne JL, Moore JH, Wagner A (2013) Robustness, Evolvability, and the Logic of Genetic Regulation. Artif Life 16: 1–16 10.1162/ARTLa00099 PMC4226432 23373974

[18] Cao H, Wu J, Wang Y, Jiang H, Zhang T, et al (2013) An integrated tool to study MHC region: accurate SNV detection and HLA genes typing in human MHC region using targeted high-throughput sequencing. PLoS One 8: e69388 10.1371/journal.pone.0069388 23894464 PMC3722289

[19] Noble JA, Erlich HA (2012) Genetics of type 1 diabetes. Cold Spring Harb Perspect Med 2: a007732 10.1101/cshperspect.a007732 22315720 PMC3253030

[20] Aguirre J, Buldú JM, Stich M, Manrubia SC (2011) Topological structure of the space of phenotypes: the case of RNA neutral networks. PLoS One 6: e26324 10.1371/journal.pone.0026324 22028856 PMC3196570

[21] Greenbury SF, Johnston IG, Louis AA, Ahnert SE (2014) A tractable genotype-phenotype map modelling the self-assembly of protein quaternary structure. J R Soc Interface 11: 20140249 10.1098/rsif.2014.0249 24718456 PMC4006268

[22] Bernstein BE, Birney E, Dunham I, Green ED, Gunter C, et al (2012) An integrated encyclopedia of DNA elements in the human genome. Nature 489: 57–74 10.1038/nature11247 22955616 PMC3439153

[23] Habegger L, Balasubramanian S, Chen DZ, Khurana E, Sboner A, et al (2012) VAT: a computational framework to functionally annotate variants in personal genomes within a cloud-computing environment. Bioinformatics 28: 2267–2269 10.1093/bioinformatics/bts368 22743228 PMC3426844

[24] Khurana E, Fu Y, Chen J, Gerstein M (2013) Interpretation of genomic variants using a unified biological network approach. PLoS Comput Biol 9: e1002886 10.1371/journal.pcbi.1002886 23505346 PMC3591262

[25] Schaffner SF, Foo C, Gabriel S, Reich D, Daly MJ, et al (2005) Calibrating a coalescent simulation of human genome sequence variation. Genome Res 15: 1576–1583 10.1101/gr.3709305 16251467 PMC1310645

[26] Alves I, Srámková Hanulová A, Foll M, Excoffier L (2012) Genomic data reveal a complex making of humans. PLoS Genet 8: e1002837 10.1371/journal.pgen.1002837 22829785 PMC3400556

[27] Messer PW, Petrov DA (2013) Population genomics of rapid adaptation by soft selective sweeps. Trends Ecol Evol 28: 659–669 10.1016/j.tree.2013.08.003 24075201 PMC3834262

[28] Durbin RM, Altshuler DL, Abecasis GR, Bentley DR, Chakravarti A, et al (2010) A map of8human genome variation from population-scale sequencing. Nature 467: 1061–1073 10.1038/nature09534 20981092 PMC3042601

[29] Dall'Olio GM, Vahdati AR, Jaume B, Andreas W, Hafid L (2014) VCF2Networks: applying Genotype Networks to Single Nucleotide Variants data. arXiv:1401.2016. Available: http://arxiv.org/abs/1401.2016. Accessed 2014 Apr 27.

[30] Danecek P, Auton A, Abecasis G, Albers CA, Banks E, et al (2011) The variant call format and VCFtools. Bioinformatics 27: 2156–2158 10.1093/bioinformatics/btr330 21653522 PMC3137218

[31] Csardi G, Nepusz T (2006) The igraph software package for complex network research. InterJournal, Complex Syst.

[32] Nei M (1987) Molecular Evolutionary Genetics. Columbia University Press, New York.

[33] Sandve GK, Nekrutenko A, Taylor J, Hovig E (2013) Ten Simple Rules for Reproducible Computational Research. PLoS Comput Biol 9: e1003285 10.1371/journal.pcbi.1003285 24204232 PMC3812051

[34] Smoot ME, Ono K, Ruscheinski J, Wang PL, Ideker T (2011) Cytoscape 2.8: new features for data integration and network visualization. Bioinformatics 27: 431–432 10.1093/bioinformatics/btq675 21149340 PMC3031041

[35] Neph S, Kuehn MS, Reynolds AP, Haugen E, Thurman RE, et al (2012) BEDOPS: high-performance genomic feature operations. Bioinformatics 28: 1919–1920 10.1093/bioinformatics/bts277 22576172 PMC3389768

[36] Quinlan AR, Hall IM (2010) BEDTools: a flexible suite of utilities for comparing genomic features. Bioinformatics 26: 841–842 10.1093/bioinformatics/btq033 20110278 PMC2832824

