# Correction: Human Genome Variation and the Concept of Genotype Networks

**DOI:** 10.1371/journal.pone.0107347

**Published:** 2014-08-27

**Authors:** The PLOS ONE Staff

There is an error in affiliation 4 for Hafid Laayouni. Affiliation 4 should be:

Department de Genètica i de Microbiologia, Grup de Biologia Evolutiva (GBE), Universitat Autonòma de Barcelona, Bellaterra (Barcelona), Spain

Additionally, Table 1 is formatted incorrectly. Please refer to the correct Table 1 here:

**Table 1:** Regions showing top scores in the genome.

region	criteria	Closest gene	Distance to closest gene	Description of closest gene	2^nd^ closest gene	Description of 2^nd^ closest gene
chr2:91959344-91968231	high number of components	GGT8P	inside gene	pseudogene		
chr6:33037767-33038449	high number of components	HLA-DPA1 / HLA-DPB1	inside gene	Homo sapiens major histocompatibility complex, class II		
chr:7203189-7420319641	high number of components	ITGB8	50,684 bp	integrin	HLA-DPA1 / HLA-DPB1	major histocompatibility complex, class II
chr5:108634323-108635534	high average degree	PJA2	34,876 bp	praja ring finger 2, E3 ubiquitin protein ligase	AK021888	unknown function
chr8:25935936-25937929	high average degree	EBF2	inside gene	early B-cell factor 2		
chr6:32507854-32508257	high average path length	HLA-DRB1	inside gene	Homo sapiens major histocompatibility complex, class II		
chr6:32568909-32569343	high average path length	HLA-DRB5	11,297 bp	Homo sapiens major histocompatibility complex, class II	HLA-DQA1	major histocompatibility complex, class II
chr6:32611264-32611586	high average path length	HLA-DQA1	inside gene	Homo sapiens major histocompatibility complex, class II		
chr3:36921415-36921688	high number of vertices	TRANK1	inside gene	tetratricopeptide repeat and ankyrin repeat Containing 1		
chr4:9176678-9178624	high number of vertices	C9JJH3	33,759 bp	Deubiquitinating enzyme	LOC650293	transmembrane helix receptor
chr8:35105546-35106981	high number of vertices	UNC5D	inside gene	receptor of netrin involved in nervous system		
chr4:9200148-9202368	few components, but large number of vertices	USP17L10	10,015 bp	Deubiquitinating enzyme		
chr6:31357915-31358747	few components, but large number of vertices	MICA	8,814 bp	MHC class I polypeptide-related sequence A	HLA-B	major histocompatibility complex, class I
chr6:31455010-31456012	few components, but large number of vertices	MICB	6,646 bp	MHC class I polypeptide-related Sequence B	uc003ntm.3	HLA complex Group 26 (non-protein coding)
