# Supplementary figures and images for: Human Genome Variation and the Concept of Genotype Networks
Source: PLoS One. 2014 Jun 9;9(6):e99424. doi: 10.1371/journal.pone.0099424 (PMC4049842; doi:10.1371/journal.pone.0099424)

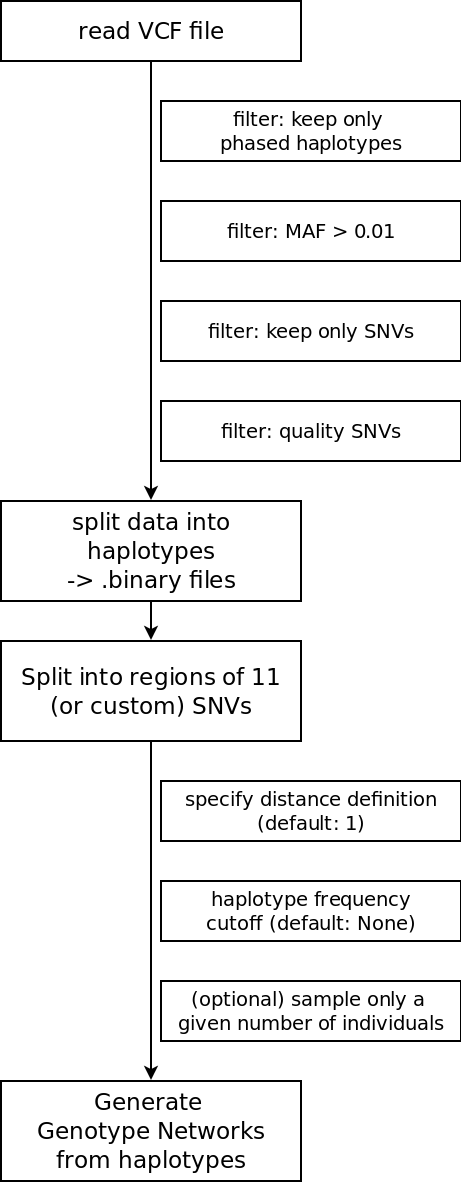

Supplement: Figure S1 — Workflow used to calculate genotype network properties from a VCF file. (PNG) [file pone.0099424.s001.png]

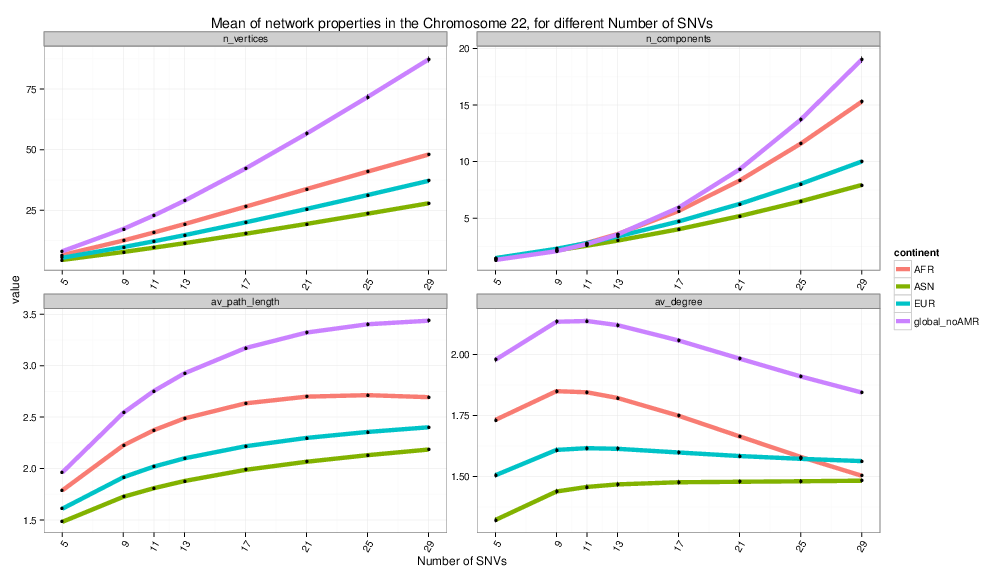

Supplement: Figure S2 — Distribution of genotype network properties in chromosome 22, changing the number of SNVs used to generate each network (window size), from 5 to 29 SNVs. In order to have the same number of individuals in each population, each point is based on 5 samples of 370 haplotypes. (PNG) [file pone.0099424.s002.png]

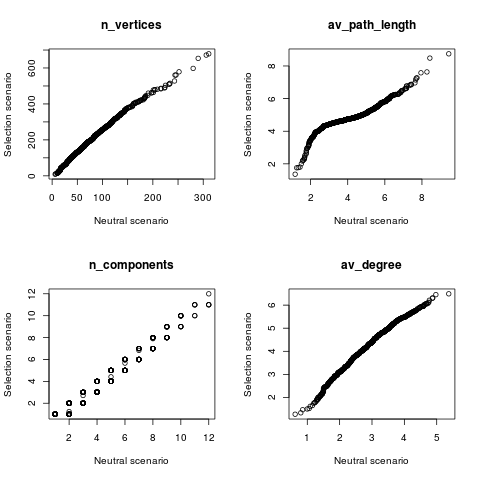

Supplement: Figure S3 — Quantile-quantile plots of neutral vs selection simulations. Only the networks of the global populations (African + European + Asians) have been included. (PNG) [file pone.0099424.s003.png]
