# Supplementary material for: Human Genome Variation and the Concept of Genotype Networks
Source: PLoS One. 2014 Jun 9;9(6):e99424. doi: 10.1371/journal.pone.0099424 (PMC4049842; doi:10.1371/journal.pone.0099424)
Supplement: Table S1 — Wilcoxon test comparing coding and non coding networks. (DOC) [file pone.0099424.s004.doc]

**Supplementary Table 1**: Wilcoxon test comparing coding and non coding networks.

| **Network Property** | **Continent** | **Mean  Coding Networks** | **Mean non-coding Networks** | **SD coding Networks** | **SD non-coding Networks** | **SE of the Mean  coding Networks** | **SE of the Mean non-coding Networks** | **p.value Wilcoxon coding vs noncoding** | **p.value Wilcoxon, Corrected Bonferroni** |
| --- | --- | --- | --- | --- | --- | --- | --- | --- | --- |
| Number of vertices | Global  (no AMR) | 26.5552 | 22.2909 | 23.4718 | 15.9832 | 0.7275 | 0.0473 | 1,05 x 10^-5^ | 1,05 x 10^-3^ |
| Number of vertices | AFR | 18.1595 | 15.5895 | 13.8822 | 9.7145 | 0.4303 | 0.0287 | 2,52 x 10^-5^ | 2,52 x 10^-3^ |
| Number of vertices | ASN | 11.3132 | 9.2384 | 10.7100 | 7.5662 | 0.3319 | 0.0224 | 4,90 x 10^-10^ | 4,90 x 10^-8^ |
| Number of vertices | EUR | 14.2305 | 11.8053 | 13.8575 | 9.2375 | 0.4295 | 0.0273 | 9,11 x 10^-5^ | 9,11 x 10^-3^ |
| Number of components | Global  (no AMR) | 2.3650 | 2.7084 | 1.1491 | 1.3327 | 0.0356 | 0.0039 | 7,60 x 10^-17^ | 7,60 x 10^-15^ |
| Number of components | AFR | 2.4409 | 2.8142 | 1.1166 | 1.3446 | 0.0346 | 0.0040 | 1,24 x 10^-18^ | 1,24 x 10^-16^ |
| Number of components | ASN | 2.4601 | 2.5714 | 1.1638 | 1.2175 | 0.0361 | 0.0036 | 3,56 x 10^-3^ | 3,56 x 10^-1^ |
| Number of components | EUR | 2.6628 | 2.8065 | 1.3332 | 1.2820 | 0.0413 | 0.0038 | 1,03 x 10^-5^ | 1,03 x 10^-3^ |
| average  path length | Global  (no AMR) | 2.8685 | 2.7457 | 0.7677 | 0.7864 | 0.0238 | 0.0023 | 2,30 x 10^-8^ | 2,30 x 10^-6^ |
| average  path length | AFR | 2.5356 | 2.3656 | 0.7465 | 0.7217 | 0.0231 | 0.0021 | 9,45 x 10^-14^ | 9,45 x 10^-12^ |
| average  path length | ASN | 1.8722 | 1.7307 | 0.7765 | 0.7282 | 0.0241 | 0.0022 | 5,79 x 10^-10^ | 5,79 x 10^-8^ |
| average  path length | EUR | 2.1187 | 1.9841 | 0.7844 | 0.7209 | 0.0243 | 0.0021 | 3,56 x 10^-7^ | 3,56 x 10^-5^ |
| average  degree | Global  (no AMR) | 2.3283 | 2.1216 | 0.7195 | 0.6192 | 0.0223 | 0.0018 | 5,31 x 10^-17^ | 5,31 x 10^-15^ |
| average  degree | AFR | 2.0392 | 1.8356 | 0.6364 | 0.5690 | 0.0197 | 0.0017 | 1,37 x 10^-21^ | 1,37 x 10^-19^ |
| average  degree | ASN | 1.6062 | 1.4382 | 0.7564 | 0.6904 | 0.0234 | 0.0020 | 3,06 x 10^-12^ | 3,06 x 10^-10^ |
| average  degree | EUR | 1.7444 | 1.5980 | 0.7503 | 0.6531 | 0.0233 | 0.0019 | 2,30 x 10^-7^ | 2,30 x 10^-5^ |

Wilcoxon test comparing the groups of “coding” and “non coding” networks. Description of the Network Property labels: n_vertices → Number of Vertices; n_components → Number of Components; av_path_lenght → Average Path Lenght; av_degree → Average Degree. Description of the Continant labels: global_noAMR → all the African, Asians and European populations.
